# Inhibitory and Antioxidant Activities of *Saccharina japonica* Extracts Processed by Subcritical Water Extraction and *Enterococcus faecalis* Fermentation

**DOI:** 10.4014/jmb.2504.04045

**Published:** 2025-08-07

**Authors:** Yong Zhao, Chae Hun Ra

**Affiliations:** Department of Food Science and Biotechnology, College of Engineering, Global K-Food Research Center, Hankyong National University, Anseong-Si 17579, Republic of Korea

**Keywords:** *Saccharina japonica*, subcritical water extraction, fermentation, lipase inhibition, antioxidant activity

## Abstract

Marine algae, notably *Saccharina japonica*, are valuable sources of essential nutrients, including carbohydrates, vitamins, and minerals, as well as bioactive compounds, such as phenols and pigments. In this study, subcritical water extraction of *S. japonica*, a brown seaweed, was conducted using a 10% (w/v) seaweed slurry at a temperature of 140°C for 10 min. The concentrations of mannitol, laminarin, and total phenolic content were determined to be 18.93 g/l, 10.79 g/l, and 1.94 mg GAE/g, respectively. The *S. japonica* fermented using *Enterococcus faecalis* (SFL) resulted in a maximum biomass production of 4.43 g dcw/l and exhibited an inhibitory activity of 78.69% against pancreatic lipase. The *S. japonica* fermented using *E. faecalis* (SFE) extracts exhibited α-glucosidase inhibitory and antioxidant activities of 59.23% and 65.60%–66.57%, respectively, at a concentration of 3.0 mg/ml. These results facilitate the development of functional seaweed food formulations derived from microbial fermentation.

## Introduction

*Saccharina japonica* is a prominent species of large brown alga that is widely distributed across the northwestern Pacific ocean [1]. It has been used as both a food and medicinal resource in East Asian countries, including China, Japan, and Korea, for over a thousand years. Since the introduction of modern cultivation techniques in the 1950s, *S. japonica* has become an economically significant crop in these regions [2]. Commonly known as sea tangle or Dasima, *S. japonica* is extensively cultivated in Korea within a well-established marine aquaculture system. This seaweed is notable for its high content of easily degradable carbohydrates, including alginate, laminarin, and mannitol, which have applications in food and natural products. Mannitol and glucose can be fermented into high-value compounds from milled seaweed. Consequently, *S. japonica* has been selected as the biomass for lactic acid bacteria (LAB) fermentation in this study. The bioactive compounds found in *S. japonica* exhibit key biological functions, including antioxidant, anti-inflammatory, antitumor, and hypoglycemic effects. These properties highlight the significant potential of *S. japonica* in developing and applying functional foods and pharmaceuticals, offering extensive opportunities for innovation in health and nutrition industries [3-7].

Methanol, ethanol, n-hexane, petroleum ether, diethyl ether, chloroform, ethyl acetate, and glycerol are the commonly used extraction solvents in laboratories because they effectively enhance extraction efficiency. However, most organic solvents are toxic and cannot be used in food processing [8]. In contrast, subcritical water extraction (SWE) employs water as the sole extraction solvent, making it non-toxic, safe, and highly suitable for extracting natural products [9]. SWE operates by heating water under pressure to temperatures above 100°C but below its critical temperature of 374°C, where it remains in a liquid state. Under subcritical conditions, changes in hydrogen bonding, ionic hydration, and the cluster structure of water enable controlled polarity shifts. This enables the efficient extraction of a broad spectrum of compounds, ranging from polar to nonpolar substances [10]. Numerous reports have been published on the use of SWE to obtain polysaccharides and phenolic compounds from seaweed [11, 12].

In addition to extraction methods, fermentation with LAB has been identified as a viable strategy for enhancing the bioactivity of polysaccharides derived from *S. japonica*. LAB fermentation facilitates the biotransformation of polysaccharides, potentially augmenting their antioxidant, anti-inflammatory, and antimicrobial properties [13-15]. In this study, we evaluated the effects SWE conditions on slurry content, temperature, and thermal hydrolysis time using the one-factor-at-a-time (OFAT) method. Additionally, we investigated the fermentation profiles, lipase inhibition, α-glucosidase inhibition, and antioxidant activities of LAB-fermented *S. japonica* hydrolysate. Our findings highlight the potential of LAB-fermented *S. japonica* extracts for developing functional food products and therapeutic agents, thus providing new opportunities for the valorization of brown algae.

## Materials and Methods

### Materials and Subcritical Water Extraction

*S. japonica* (sea tangle; Dasima) was obtained from Gijang, a local food company in Busan, Korea. The reagents used for this research were of HPLC grade, and the standards were purchased from Sigma-Aldrich (USA). The fresh seaweed was washed thoroughly to remove impurities, dried in the sun, and cut into uniform squares measuring 2 cm × 2 cm. Samples weighing 30, 40, 50, and 60 g were individually placed in an accelerated solvent extractor (R-401 dual-critical extraction system, Chemresys, Republic of Korea), with 500 ml of distilled water introduced into the reactor. The reactor was sealed and the pressure was adjusted to 3 MPa using nitrogen gas. The mixture was then heated using an electric heater and the stirring speed was set to 100 rpm. The SWE conditions were varied by setting extraction temperatures at 120, 130, 140, and 150°C, with durations of 5, 10, 15, and 20 min. A single-variable approach (OFAT) was used to assess the effects of each parameter while keeping the other conditions constant. Approximately 500 ml of the extract was obtained for each condition. All extracts were stored at –20°C to preserve bioactive compounds for further analysis.

### Total Phenolic Contents (TPC)

The total phenolic content was measured using a modified Folin–Ciocalteu reagent method. Briefly, 0.1 ml of pretreated extracts was mixed with 2 ml of 2% sodium carbonate (Na_2_CO_3_) and vortexed for 3 min. Subsequently, 0.1 ml of 50% Folin–Ciocalteu reagent was added, and the mixture was incubated at room temperature for 30 min. The absorbance of the mixture at 700 nm was measured and compared with a standard calibration curve of gallic acid. The results were expressed as milligrams of gallic acid equivalent (GAE) per gram of dried *S. japonica* mass (mg GAE/g).

### Lactic Acid Bacteria Fermentation

Four strains of LAB were obtained from the Korean Culture Collection of Probiotics (KCCP, Republic of Korea), including *Lactobacillus johnsonii* HDL 203, *Lactobacillus rhamnosus* HWL201, *Leuconostoc mesenteroides* SGL 152, and *Leuconostoc citreum* CKS 001. *Enterococcus faecalis* RP-004 was obtained from Raphas Co., Ltd.(Republic of Korea). These strains served as seed cultures in De Man, Rogosa, and Sharpe (MRS) broth, incubated at 30°C for 24 h in a shaking incubator. Nutrient supplements of 20 g/l glucose, 10 g/l yeast extract, 5 g/l K_2_HPO_4_, 0.25 g/l MgSO_4_, and 1 g/l ascorbic acid (vitamin C) were added to the *S. japonica* hydrolysate-modified synthetic medium. Subsequently, 5% (v/v) inoculates were transferred to 250 ml Erlenmeyer flasks containing 100 ml of *S. japonica* hydrolysate. The seaweed hydrolysates were fermented at 30°C and 200 rpm with five LAB strains. Samples were obtained periodically to determine the optical density (OD_600_), laminarin, residual sugars, acetic acid, and lactic acid concentrations and stored at –20°C prior to analysis.

### Pancreatic Lipase Inhibition

The method for determining lipase inhibitory activity by Kim *et al*. [16] was modified. Porcine pancreatic lipase was used for lipase inhibition tests of fermentation cell pellets (FCP, MRS medium), fermentation broth supernatant (FBS, MRS medium), and *S. japonica* fermented using LAB (SFL, fermentation supernatant). FCP and FBS were prepared in MRS medium cultures for 72 h using five LAB strains, including *Lactobacillus johnsonii* HDL 203, *L. rhamnosus* HWL201, *E. faecalis* RP-004, *Leuconostoc mesenteroides* SGL 152, and *Leuconostoc citreum* CKS 001. The cells were harvested by centrifugation at 1,390 ×*g* for 10 min, and the residual medium (supernatant) was collected for the FBS samples. The collected cells intended for FCP samples were thoroughly washed with distilled water and resuspended in phosphate-buffered saline (PBS; pH 7.0). The *S. japonica* hydrolysates obtained under optimal conditions for SWE were fermented under the same conditions using SFL samples. One unit of enzyme was required to release 1 mmol of *p*-nitrophenol under standard assay conditions. Briefly, the enzyme-buffer solution was prepared by adding 30 μl of lipase solution (in 10 mM MOPS and 1 mM EDTA, pH 6.8) to 850 μl of Tris buffer (100 mM Tris-HCl and 5 mM CaCl_2_, pH 7.0). After mixing 100 μl of the sample extracts with 880 μl of enzyme-buffer solution, the enzyme-sample extract mixture was incubated for 15 min at 37°C. Subsequently, 20 μl of the substrate solution (10 mM p-NPB in dimethyl formamide) was added. Enzymatic reactions were conducted for 15 min at 37°C and measured at 405 nm using a spectrophotometer. Orlistat was used as the positive control.

### α-Glucosidase Inhibition

Fermentation was performed for 72 h using MRS medium and modified *S. japonica* hydrolysate under optimized conditions. The samples obtained included *E. faecalis* cell pellets (ECP, from MRS medium), *E. faecalis* fermentation supernatants (EFS, from MRS medium), and *S. japonica* fermented using *E. faecalis* (SFE, from fermentation broth). These samples were lyophilized using a freeze dryer and stored at –20°C prior to analysis. Dried ground samples of ECP, EFS, and SFE (3 g each) were extracted with 100 ml of 80% (v/v) ethanol at 60°C for 2 h. The solution was filtered through 8 μm filter paper (Whatman, UK) and concentrated at 40°C using a rotary vacuum evaporator. The extracts were dissolved in 30 ml of 50 mM potassium phosphate buffer (pH 6.8) for further experiments. The α-glucosidase inhibitory activity by Kwun *et al*. [17] was performed with slight modification. All extracts of 50 μl were mixed with 50 μl of α-glucosidase solution. After 30 min at 37°C, 100 μl of 3 mM pNPG was added and incubated for another 30 min. The reaction was stopped with 750 μl of 0.1 M Na_2_CO_3_, and absorbance was measured at 405 nm using a microplate reader (Multiskan FC, Thermo Fisher Scientific, USA). Acarbose was used as the positive control at a concentration of 0.4 mg/ml. The percentage of α-glucosidase inhibition was calculated using Eq. (1):

$$\text{α-Glucosidase inhibition=}\left[\text{1-}\left(\frac{\left(\text{Abssample-Absblank}\right)}{\left(\text{Abscontrol}\right)}\right)\right]\text{×100}$$ (1)

### DPPH and ABTS Assays

The antioxidant abilities of the ECP, EFS, and SFE extracts were assessed using DPPH and ABTS radical scavenging activity assays. DPPH (2,2-diphenyl-1-picrylhydrazyl) solutions were prepared by dissolving 0.4 mM DPPH in 95% (v/v) ethanol until the absorbance at 517 nm was 0.94–0.97. The samples (10 μl) were mixed with 190 μl DPPH solution and stored in complete darkness for 30 min at room temperature (RT). After incubation, the absorbance was measured at 517 nm using a microplate reader.

The ABTS solution was prepared by mixing 7.4 mM ABTS with 2.6 mM potassium persulfate and letting the mixture react in the dark at RT for 24 h before use. The solution was then diluted with phosphate-buffered saline (pH 7.4) to achieve an absorbance of 0.700 ± 0.020 at 732 nm. For the assay, 50 μl of each extract was mixed with 950 μl of the diluted ABTS solution and allowed to react for 10 min at RT. Absorbance was measured at 732 nm using a microplate reader. Ascorbic acid (1 mg/ml) was used as a positive control. DPPH and ABTS radical scavenging activities (%) were calculated using Eq. (2).

$$\text{Radical scavenging activity (\%) =}\left(\text{1-}\left[{\text{Abs}}_{\text{sample}}\right]\text{/}\left[{\text{Abs}}_{\text{blank}}\right]\right)\text{×100}$$ (2)

where Abs_sample_ is the absorbance of the sample mixed with the DPPH/ABTS solution, and Abs_blank_ is the absorbance of the sample solvent-mixed DPPH/ABTS solution.

### Analytical Methods

The cell growth was monitored by measuring OD_600_ using a UV–visible spectrophotometer (EMC-18PC-UV, EMCLAB, Germany). The value from OD_600_ was converted into cell concentration using a standard curve that relates OD_600_ to the dry cell weight. Specifically, a value of OD_600_ = 1.0 corresponds to the following dry cell weights: 0.36 g dcw/l for *L. johnsonii*, 0.73 g dcw/l for *L. rhamnosus*, 0.66 g dcw/l for *E. faecalis*, 1.15 g dcw/l for *L. mesenteroides*, and 0.35 g dcw/l for *L. citreum*.

Laminarin was purified as described by Rajauria *et al*. [18]. Briefly, the sample (1 ml) was mixed with 100%ethanol in a 1:4 (v/v) ratio and centrifuged at 994 ×*g* for 5 min. The supernatant was discarded, and the pellet was dissolved in some ultrapure water and left overnight at 4°C. The suspension was then mixed with 3 ml of calcium chloride (2%, w/v) and centrifuged at 994 ×*g* for 5 min. The supernatant was analyzed using a high-performance liquid chromatography (HPLC) system (Agilent 1200 Series; Agilent Inc., USA) equipped with a refractive index detector (RID). The residual sugar (glucose and mannitol), acetic acid, and lactic acid concentrations were determined using the HPLC system equipped with an RID. The Bio-Red Aminex HPX-87H column (300.0 × 7.8 mm) was maintained at 65°C, and the samples were eluted with 5 mM H_2_SO_4_ at 0.6 ml/min. Each sample was analyzed in triplicate, and the mean values were calculated. All the chemicals and reagents used were of analytical grade.

### Statistical Analysis

Each experiment was performed in triplicate. Statistical significance was assessed using a one-way analysis of variance (ANOVA), followed by Duncan’s multiple range test (*P* < 0.05) using SPSS version 23 (SPSS, USA).

## Results and Discussion

### Evaluation of Subcritical Water Extraction Conditions for *Saccharina japonica*

**Fig. 1:**
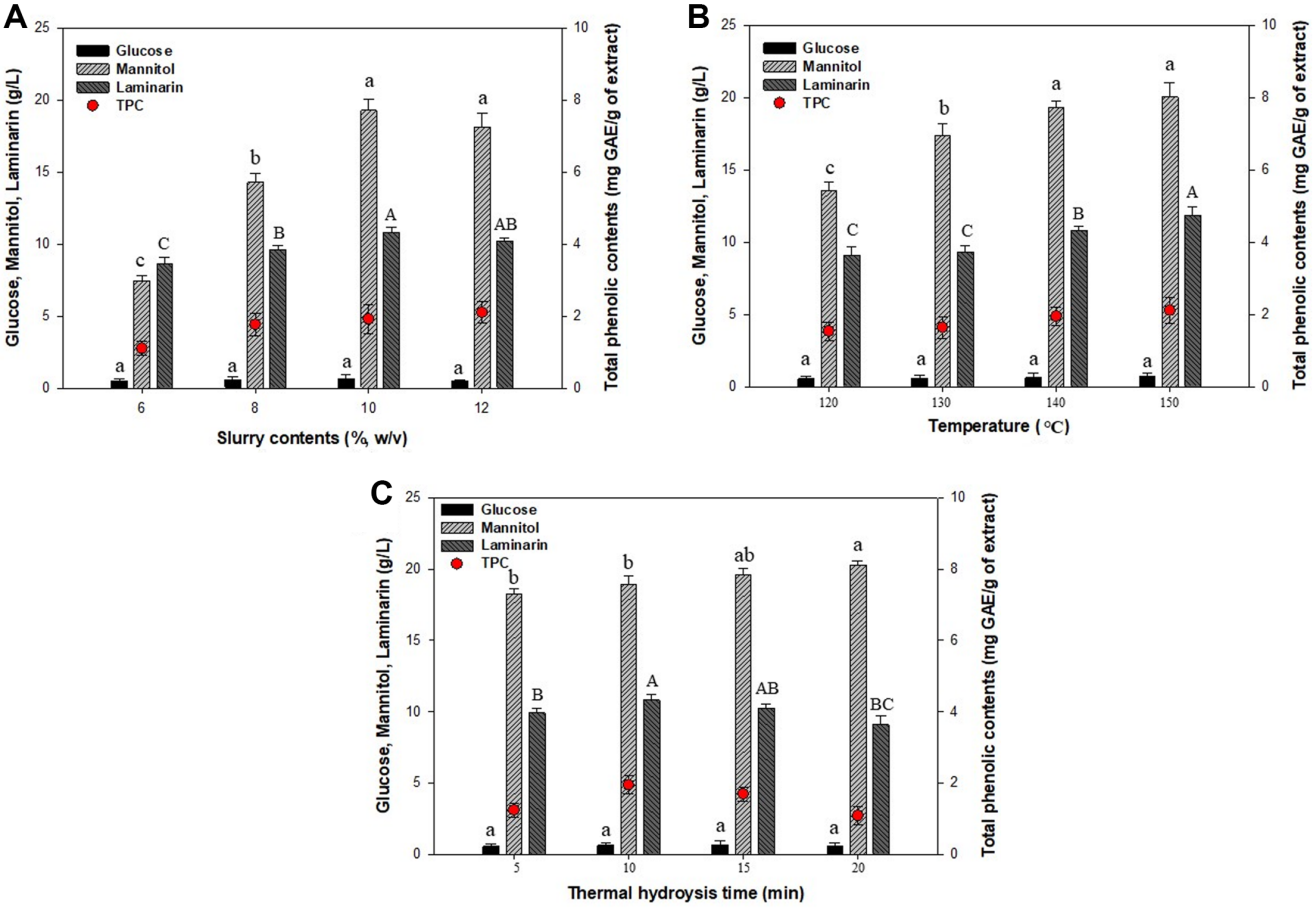
Evaluation of the subcritical water extraction (SWE) conditions based on (A) slurry content, (B) Temperature, and (C) thermal hydrolysis time. The initial SWE conditions were set at a temperature of 140°C and a thermal hydrolysis time of 10 min. Data are expressed in terms of mean ± SD. Different small letters indicate significant differences (*P* < 0.05, Duncan’s test).

SWE conditions were evaluated using a seaweed slurry of 6%–12% (w/v), temperature of 120–150°C, and thermal hydrolysis time of 5–20 min, as shown in Fig. 1. The effects of SWE conditions at temperature of 140°C and thermal hydrolysis time of 10 min with various slurry contents are shown in Fig. 1A. Subcritical water extracts were analyzed to quantitatively determine the concentrations of laminarin, mannitol, glucose, and TPC. Increasing the slurry content resulted in a proportional increase in the monosaccharide and TPC concentrations, with the exception of glucose. After 10 min at 140°C with a 12% (w/v) slurry content, the highest concentrations obtained were 19.29 g/l for mannitol, 10.79 g/l for laminarin, and 0.66 g/l for glucose. The TPC of 10%(w/v) seaweed slurry exhibited an appropriate concentration (1.92 mg GAE/g), whereas an increase in the slurry content to 12% resulted in a slight increase in TPC (2.10 mg GAE/g). A 10 % (w/v) seaweed slurry was used in subsequent experiments. However, a further increase in the slurry content over 10% (w/v) using SWE resulted in a decrease in mannitol and laminarin concentrations. These results indicate that as the solid-to-liquid (S/L) ratio increased, the solution became saturated with the solute. This saturation can affect the mass transfer rate, prevent dissolution of the solid into the solution, and decrease the yield. Saravana *et al*. [19] found that the S/L ratio significantly affects the subcritical water extraction of crude fucoidan from *S. japonica*. They observed that when the S/L ratio exceeded 0.07 g/ml, the yield began to decline. In contrast, the presence of an agitator in the extractor can enhance mass transfer; higher agitation speeds contribute to improving mass transfer but may also increase degradation rates [20].

As shown in Figure 1B, the effects of different temperatures were evaluated using a 10% (w/v) seaweed slurry over a hydrolysis duration of 10 min. An increase in temperature from 140 to 150°C resulted in elevated concentrations of mannitol, laminarin, and TPC. This indicated that temperature substantially influenced the efficiency of extracts from *S. japonica*.

As the temperature was further increased to 140–150°C, the concentrations of mannitol, laminarin, and TPC were observed to increase slightly, reaching values of 19.29– 20.03 g/l, 10.79– 11.84 g/l, and 1.94– 2.11 mg GAE/g, respectively. Thus, a temperature of 140°C was selected as the suitable setting for this study. In contrast, the opposite effect was observed in the root of the brown seaweed *S. japonica*, where the highest recorded SWE efficiency was 77.07% at 230°C [21]. Additionally, we observed that the total phenolic and flavonoid content may decrease when the extraction temperature exceeds 200°C. Total and reducing sugars tend to increase as the extraction temperature rises from 110 to 170°C before decreasing again. The lower extraction temperature observed in this study may be owing to the use of whole dried *S. japonica* seaweed without grinding, which could have hindered hydrolysis.

The effects of various subcritical water hydrolysis durations were evaluated using a 10% (w/v) slurry at a reaction temperature of 140°C, as shown in Figure 1C. Our results showed that the mannitol concentration slightly increased with increasing hydrolysis time. The highest concentration of mannitol, 20.28 g/l, was achieved using SWE for 20 min. However, laminarin and TPC gradually decreased with a further increase from 10 to 20 min. These results indicate that an extended extraction time may adversely affect the yields of laminarin and TPC, owing to the potential degradation of the extract compounds. These results are similar to those reported by Fan *et al*. [22] and Dobrinčić *et al*. [23]. They reported that longer extraction times could lead to the degradation of bioactive compounds and algal polysaccharides, resulting in reduced yields. Based on the conditions under which mannitol, laminarin, and TPC concentrations were obtained, a hydrolysis time of 10 min was used in this study. Therefore, the best conditions for SWE were a 10% (w/v) slurry content at 140°C for 10 min.

### Growth of LAB Strains and Their Effects on Lipase Inhibition

**Fig. 2:**
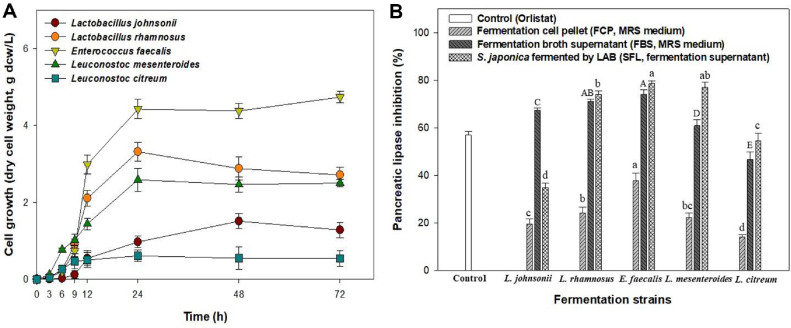
Comparison of five LAB fermentations regarding (A) cell growth and (B) pancreatic lipase inhibition. Data are expressed in terms of mean ± SD. Different small letters indicate significant differences (*P* < 0.05, Duncan’s test).

The fermentation profiles of the five distinct LAB strains were obtained and compared in terms of cell growth and lipase inhibition. As shown in Fig. 2A, pure cultures of *L. johnsonii*, *L. rhamnosus*, *E. faecalis*, *Leu. mesenteroides*, and *Leu. citreum* was evaluated using MRS medium at 30°C and 200 rpm for 72 h. The growth of the five LAB strains reached the stationary phase within 24-48 h. When the pH dropped below 4.0 at 72 h, cell growth ceased and lactic acid synthesis was inhibited (data not shown). Among them, *E. faecalis* showed the highest cell concentration at 4.43 g dcw/l for 24 h, followed by *L. rhamnosus* (3.31 g dcw/l for 24 h), *L. mesenteroides* (2.58 g dcw/l for 24 h), *L. johnsonii* (1.51 g dcw/l for 48 h), and *L. citreum* (0.61 g dcw/l for 24 h). Mehmeti *et al*. [24] found that the lactate flux in *E. faecalis* increased significantly with the growth rate. The gene levels of *ldh-1* transcript increased with growth rate, with fast-growing cells showing approximately 1,700 times more *ldh-1* transcripts than slow-growing cells.

Figure 2B shows the lipase inhibitory activities of the fermentation cell pellet (FCP, MRS medium for 72 h), fermentation broth supernatant (FBS, MRS medium for 72 h), and *S. japonica* fermented by LAB (SFL, fermentation supernatant for 72 h) determined using an *in vitro* assay with porcine lipase. The inhibition of pancreatic lipase using the five LAB strains was more significant in the FBS and SFL groups than in the FCP group. Among the five LAB strains, *E. faecalis* from SFL showed the highest inhibitory activity against pancreatic lipase (78.69%), followed by *Leu. mesenteroides* (77.14%) and *L. rhamnosus* (74.10%). These results showed that the inhibition of pancreatic lipase enzyme activity was significantly increased by *S. japonica* fermented by the three strains compared to the other groups. Li *et al*. [25] also reported similar results. *Lactobacillus delbrueckii* fermented supernatant with the red seaweed *B. fusco-purpurea* (DF) showed the highest pancreatic lipase inhibitory activity at 93.48%, followed by *L. plantarum* fermented supernatant (PF) at 82.32%, unfermented red seaweed (NF) at 61.29%, *L. delbrueckii* (LD) without red seaweed at 52.52%, and *L. plantarum* (LP) without red seaweed at 34.31%. This suggests that fermented supernatants from both the DF and PF groups served as mixed inhibitors of pancreatic lipase. The presence of *Enterococcus faecalis* and *Leu. mesenteroides* are essential for the intestinal microbiota balance, metabolic syndrome mitigation, and immune response modulation. It is also effective in managing hyperlipidemia, obesity, and fatty liver disease [26-28]. Fermentation profiles of *E. faecalis* and *Leu. mesenteroides* were carried out in *S. japonica* hydrolysates.

### Fermentation Using *E. faecalis* and *Leu. mesenteroides* with *S. japonica* Hydrolysate

**Fig. 3:**
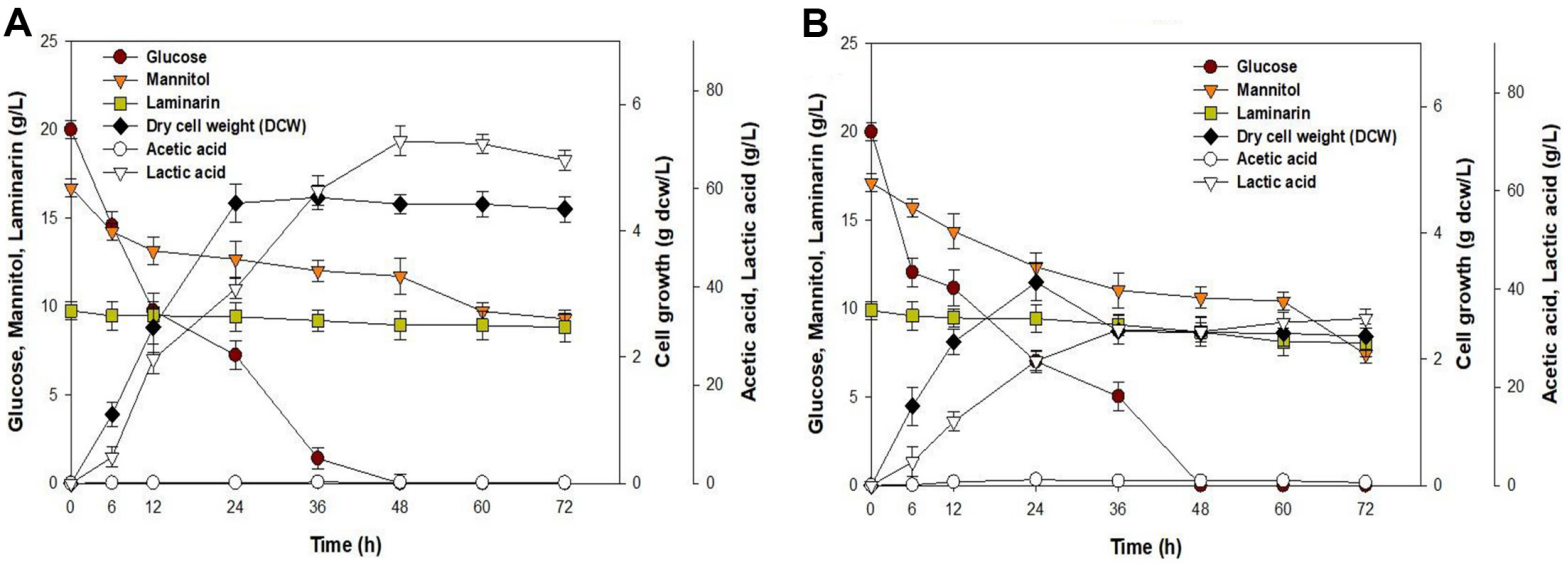
Fermentation profiles using (A) *E. faecalis* and (B) *Leu. mesenteroides* with *S. japonica* hydrolysate medium. The seaweed hydrolysates were fermented at a temperature of 30°C and agitated at 200 rpm. Data are expressed in terms of mean ± SD.

LAB fermentation was conducted using *E. faecalis* (A) and *Leu. mesenteroides* (B) using a hydrolysate medium from *S. japonica* prepared through SWE under optimal conditions, as shown in Figure 3. The fermentation profiles indicated the uptake of monosaccharides (glucose and mannitol), laminarin concentration, cell growth, and production of organic acids (acetic and lactic acid) using two LAB strains over a period of 72 h.

Figure 3A shows that glucose was completely consumed within 48 h, whereas approximately 9.30 g/l of mannitol remained unutilized by the end of the fermentation period. The growth of *E. faecalis* resulted in a maximum biomass production of 4.52 g dcw/l, reaching the stationary phase at 24 h. Additionally, the lactic acid concentration increased to 69.70 g/l before decreasing to 65.78 g/l by the end of the fermentation. The maximum acetic acid concentration increased by 0.24 g/l at 36 h after the acetic acid level had decreased to nearly zero during fermentation. Additionally, the laminarin concentration achieved 9.76 g/l under optimal SWE conditions, and this level remained stable throughout the fermentation process. *E. faecalis* has been extensively used in the processing and production of fermented dairy products owing to its remarkable potential for pathogenicity and functional food applications [29, 30]. These results suggest that Enterococci fermentation enhances the release of bioactive compounds from the strain itself or from seaweeds, thus increasing their biological activities.

Figure 3B shows that the initial 0–24 h corresponded to the exponential phase of cell growth for *Leu. mesenteroides*, during which lactic acid was produced. The maximum concentration of cells was 3.21 g dcw/l at 24 h, and lactic acid reached 34.0 g/l by 72 h. Glucose was consumed within 48 h, whereas mannitol levels remained until 72 h, indicating LAB's preference for glucose and less efficient consumption of mannitol. This phenomenon resembles the inhibitory effect of glucose on the metabolic degradation of xylose in lignocellulosic hydrolysates, emphasizing a critical aspect of metabolic processes that requires further investigation. Carbon catabolite repression (CCR) of glucose on mannitol has been observed in various microorganisms, including *B. subtilis*, *Bacillus licheniformis*, *Escherichia coli*, and *Saccharomyces cerevisiae* [31]. Thus, we selected *E. faecalis* as the best strain for α-glucosidase inhibition and the antioxidant capacity of LAB-fermented *S. japonica* hydrolysate.

### α-Glucosidase Inhibitory Activity of *E. faecalis*-Fermented *S. japonica* Hydrolysate

**Fig. 4:**
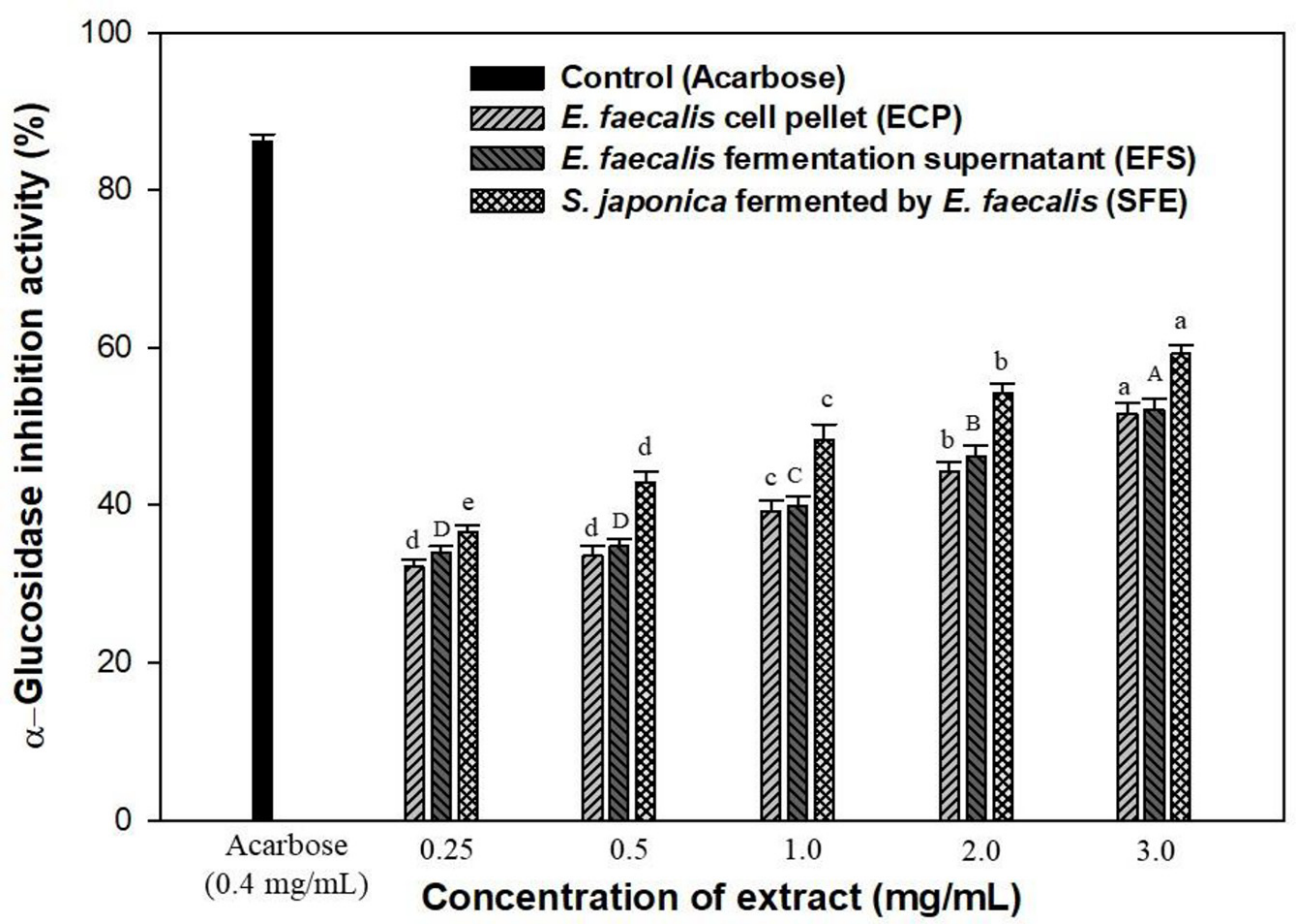
Effect of ECP, EFS, and SFE extracts on α-glucosidase inhibitory activity. Acarbose was used as a positive control. Data are expressed in terms of mean ± SD. Different small letters indicate significant differences (*P* < 0.05, Duncan’s test).

Crude ethanolic extracts of ECP, EFS, and SFE were evaluated for their α-glucosidase inhibitory activity at concentrations ranging from 0.25 to 3.0 mg/ml, as shown in Fig. 4.

The ECP, EFS, and SFE extracts exhibited α-glucosidase inhibitory activities of 51.62%, 52.19%, and 59.23% at a concentration of 3.0 mg/ml, respectively. However, the inhibitory effect on α-glucosidase was lower than that of acarbose at a concentration of 0.4 mg/ml. The SFE extract contained a higher concentration of bioactive ingredients than the ECP and EFS extracts, suggesting that fermentation may enhance these compounds in seaweeds. Similarly, Graham *et al*. [32] reported that milk fermented with *E. faecalis* DPC5154 showed the highest level of α-glucosidase inhibitory activity at 33.41%. This result suggests that *E. faecalis* fermented milk products may benefit the management of type-2 diabetes by slowing the rapid uptake of glucose and subsequently reducing post-prandial hyperglycemia. Cai *et al*. [33] later reported that *E. faecalis* EF-1 was effective both *in vitro* and *in vivo*. This strain inhibits obesity induced by a high-fat diet (HFD) by regulating the gut microbiota and enhancing the production of short-chain fatty acids (SCFAs). Therefore, SFE extract appears to be a highly promising candidate for anti-obesity therapeutics and functional foods.

### Antioxidant Activities of *E. faecalis*-Fermented *S. japonica* Hydrolysate

**Fig. 5:**
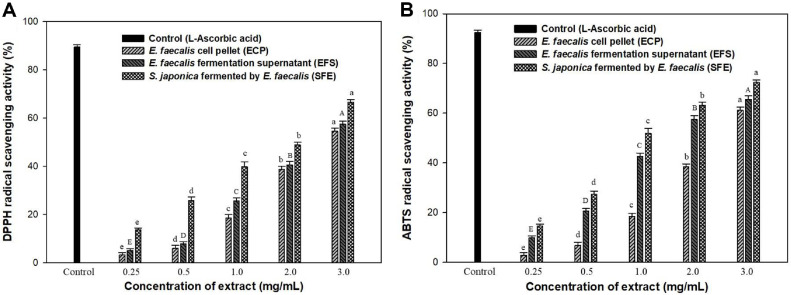
Effect of ECP, EFS, and SFE extracts on the antioxidant activities using DPPH and ABTS assays. Ascorbic acid was used as a positive control. Data are expressed in terms of mean ± SD. Different small letters indicate significant differences (*P* < 0.05, Duncan’s test).

Antioxidant activity is influenced by various factors, making it difficult to use a single model to represent the capacity of all samples. Thus, the antioxidant activities of the ECP, EFS, and SFE were evaluated using the DPPH and ABTS assays, as shown in Fig. 1. DPPH and ABTS assays measure radical scavenging processes that involve electron transfer, resulting in a color change. DPPH radical scavenging mainly interacts with lipophilic substances, whereas ABTS^+^ radical scavenging interacts with both lipophilic and hydrophilic substances. Fig. 5A shows that the DPPH scavenging ability of the ECP, EFS, and SFE increased at higher concentrations. Among the extracts samples, SFE exhibited superior antioxidant properties to the other two extracts. The highest DPPH values were observed at 3.0 mg/l, with values of 54.54% (ECP), 57.45% (EFS), and 66.57% (SFE). The results showed that the fermentates of *S. japonica* and *E. faecalis* exhibited a synergistic effect compared with single extract treatments. Afrin *et al*. [34] reported that the EC_50_ value for the DPPH radical scavenging activity of the various seaweed solvent extracts varied between 1.82 to 5.09 mg/ml. This result suggests that the SFE extract may serve as an effective antioxidant ingredient compared with the EC_50_ value of the seaweed solvent extract.

ABTS radical scavenging activity was measured to evaluate the antioxidant potential of ECP, EFS, and SFE extracts at concentrations ranging from 0.25 to 3.0 mg/ml, as shown in Figure 5B. At a concentration of 3.0 mg/ml, the SFE extract exhibited 72.37% antioxidative activity, which was significantly higher than that of the ECP (61.18%) and EFS (65.60%) extracts. This indicated that the SFE enhanced antioxidative effects, whereas fermentation did not suppress these properties. Furthermore, *E. faecalis* can mitigate the risk posed by pathogenic microbes by producing lactic acid and a range of antimicrobial peptides [32]. Lee *et al*. [35] reported that the fermentation of the brown alga *Sargassum siliquastrum* using *Lactobacillus* sp. SH-1 significantly increases the levels of total polyphenols and flavonoids. This fermentation process also enhances both DPPH radical scavenging activity and angiotensin-converting enzyme (ACE) inhibitory activity compared with non-fermented conditions. Therefore, this experimental evidence demonstrates that *Lactobacillus* fermentation not only improves the release of bioactive compounds from seaweeds but may also produce new bioactive substances, thereby enhancing their biological activities.

Furthermore, the anti-obesity effects of LAB involve several mechanisms, including adjustment of the gut microbiota [36], enhancement of SCFA production [37], modification of lipid metabolism-related gene expression [36], and reduction of inflammatory responses [38]. Heat-killed *E. faecalis* EF-2001 has been shown to reduce lipid accumulation and liver injury in mice with HFD-induced obesity by activating the AMPK signaling pathway [39]. Consequently, the SFE extract may serve as a promising candidate for health foods, nutritional supplements, and functional foods.

The findings of this study highlight the potential of *S. japonica* subcritical water extracts as an effective fermentation substrate for LAB. The suitable conditions for subcritical water extraction (SWE) were determined to be 10% (w/v) slurry content, 140°C extraction temperature, and a 10 min thermal hydrolysis, based on the concentrations of mannitol, laminarin, and TPC. The *S. japonica* hydrolysate served as a fermentation substrate for five LAB strains: *L. johnsonii*, *L. rhamnosus*, *E. faecalis*, *Leu. mesenteroides*, and *Leu. citreum*. Among the tested strains, *E. faecalis*, *Leu. mesenteroides*, and *L. rhamnosus* exhibited rapid growth rates and significant lipase inhibitory activity. Crude ethanolic extracts from *E. faecalis* cell pellets (ECP), *E. faecalis* fermentation supernatant (EFS), and *S. japonica* fermented by *E. faecalis* (SFE) demonstrated high α-glucosidase inhibitory activity and strong antioxidant properties, as measured by DPPH and ABTS assays. These results indicate that the processes of SWE and LAB fermentation enhances the biofunctional properties of seaweed, providing a promising strategy for developing functional food ingredients with potential anti-obesity, anti-diabetic, and antioxidant benefits.
